# Scalable multi-objective genetic algorithm for quantum circuit optimization

**DOI:** 10.1038/s41598-026-47674-5

**Published:** 2026-04-18

**Authors:** Roumaissa Ghlib, Rania Bouhadouza, Faicel Hnaien

**Affiliations:** 1 École Nationale Supérieure d’Informatique (ESI), Oued Smar, BP 68M, Algiers, 16309 Algeria; 2 Université de Technologie de Troyes (UTT), 12 Rue Marie Curie, Troyes, 10004 France

**Keywords:** Quantum computing, NSGA-II, Multi-objective optimization, Circuit synthesis, Fidelity, Circuit optimization, Engineering, Mathematics and computing, Physics

## Abstract

Designing compact and efficient quantum circuits that are compatible with Noisy Intermediate-Scale Quantum (NISQ) hardware remains a central challenge in quantum computing. Most existing optimization approaches rely on fidelity-based fitness functions that require computing the full unitary matrix of the circuit. However, this quickly becomes computationally intractable beyond 10–12 qubits due to the exponential memory and time complexity $O(2^{2n})$. In this work, we propose a scalable multi-objective genetic algorithm for quantum circuit optimization tailored to NISQ devices. Although evolutionary algorithms have demonstrated strong potential for circuit synthesis, current methods generally depend on full-unitary fidelity evaluation, which severely limits scalability. To address this bottleneck, we introduce two complementary strategies: (1) an independent block-based evaluation using graph partitioning, and (2) an overlapping sliding-window decomposition approach. Both strategies reduce computational complexity from $O(2^{2n})$ to $O(2^{2k})$, where $k \ll n$, making it possible to optimize circuits with more than 20 qubits in practice. Our methods are integrated into the NSGA-II multi-objective framework, enabling simultaneous optimization of fidelity, circuit depth, and gate cost, while maintaining structural compatibility with NISQ hardware–without requiring full-unitary simulation. Experimental results on benchmark circuits demonstrate high fidelity (above 0.94 for small circuits up to 8 qubits, above 0.85 for medium-scale circuits of 10–16 qubits, and above 0.80 for large-scale circuits up to 32 qubits), up to $45\%$ reduction in circuit depth, and a $10\times$ speedup compared to exact evaluation at 14 qubits.

## Introduction

Combinatorial optimization problems in fields like logistics, finance, and computational biology are increasingly approaching the computational limits of classical methods. Many of these problems are NP-hard, with solution spaces that grow exponentially, making traditional algorithms–despite sophisticated heuristics–increasingly difficult to scale efficiently.

Quantum computing promises exponential speedups for combinatorial optimization in logistics, finance, drug discovery, and computational biology. However, realizing this potential on today’s Noisy Intermediate-Scale Quantum (NISQ) devices requires circuits that are simultaneously accurate, compact, and hardware-compatible.

Quantum computing introduces a new paradigm by leveraging principles such as superposition and entanglement^1^. In addition to hybrid approaches, purely quantum algorithms such as Grover’s search algorithm^2^ and quantum amplitude amplification^3^ offer provable quadratic speedups for unstructured search and certain combinatorial problems. However, these fully quantum approaches typically require deep circuits and substantial qubit resources, particularly for implementing the problem and diffusion oracles, which can lead to significant gate overhead and energy consumption on near-term hardware. Hybrid algorithms like the Quantum Approximate Optimization Algorithm (QAOA)^4^ and the Variational Quantum Eigensolver (VQE)^5^ combine quantum circuits with classical optimizers to solve such problems, making them well-suited for today’s Noisy Intermediate-Scale Quantum (NISQ) hardware^6^.

However, running quantum algorithms on real hardware is challenging. NISQ devices are limited by gate fidelity, qubit connectivity, and decoherence, making deep or gate-heavy circuits impractical. Multi-qubit gates in particular, like CNOT, significantly reduce overall fidelity^7–10^, requiring careful trade-offs in circuit design.

To address these challenges, evolutionary algorithms–especially genetic algorithms–have gained interest for automatically generating quantum circuits that balance fidelity, gate count, and depth^11–13^. Yet most rely on evaluating full unitary matrices, which becomes computationally infeasible beyond 10–12 qubits due to exponential scaling.

Beyond evolutionary methods, a rich body of compiler-level research addresses circuit optimization through qubit mapping and routing^14^. These compilers transform logical circuits into hardware-executable ones by minimizing costly SWAP gate insertions or ion shuttling operations, depending on the target platform. While such methods excel at structural adaptation to hardware topology, they typically optimize a single cost function (e.g., gate count or depth) and do not incorporate fidelity awareness in a multi-objective sense. Our work is complementary to this line of research: rather than replacing hardware-level compilation, we address the upstream problem of scalable fidelity-guided circuit synthesis, which remains an open challenge beyond 12 qubits.

A major bottleneck nevertheless persists: current fidelity-driven optimization techniques require computing the full unitary representation, which entails exponential $O(2^{2n})$ memory and time complexity. This scalability barrier significantly restricts the application of fidelity-aware circuit optimization to larger-scale quantum systems. To overcome this scalability barrier while preserving fidelity awareness, this work makes the following key contributions:

- We present a scalable quantum circuit optimization framework built on NSGA-II that extends fidelity-aware optimization beyond the 12-qubit limit.
- We introduce two novel scalable fidelity evaluation methods–block-based partitioning and overlapping sliding windows–that avoid full unitary computation while maintaining relative ordering accuracy for evolutionary selection.
- We embed these fidelity strategies into a Pareto-based NSGA-II optimization loop that jointly minimizes fidelity error, circuit depth, and gate cost.
- We validate the framework on a diverse set of circuits, including random circuits and problem-oriented instances such as QAOA and VQE, demonstrating improved scalability and enhanced hardware-aware circuit design quality.
- By reducing complexity from $O(2^{2n})$ to $O(2^{2k})$ where $k \ll n$, our framework enables practical fidelity-guided circuit optimization for systems with 20+ qubits, opening new possibilities for deploying quantum algorithms on near-term hardware.

Unlike compiler-based local optimizers such as OAC^15^, which guarantee segment optimality with linear oracle call complexity but operate on a single cost function, our framework simultaneously optimizes fidelity, depth, and gate cost within a multi-objective Pareto framework, while avoiding full unitary computation beyond 12 qubits.

It is important to emphasize that the novelty of this work does not lie in the use of NSGA-II itself, which has already been explored in the context of quantum circuit optimization. Instead, the core contribution is the reformulation of fidelity evaluation within a multi-objective evolutionary framework. By introducing scalable, structure-aware fidelity surrogates that avoid global unitary computation, this work enables fidelity-guided optimization beyond the regime where exact evaluation is tractable.

## Background

### Quantum computation preliminaries

This section reviews fundamental quantum computing concepts essential for understanding our optimization framework.

A complex Hilbert space of dimension $2^n$ serves as the mathematical foundation of a quantum system composed of *n* qubits. The state of such a system is a unit vector $|\psi \rangle$ satisfying $\langle \psi | \psi \rangle = 1$. The Dirac notation is used throughout, where $\langle \psi |$ denotes the conjugate transpose of $|\psi \rangle$, and their inner product $\langle \psi | \psi \rangle$ is always equal to 1.

The computational basis states for a multi-qubit system are given by tensor products of single-qubit basis vectors. For example, the two-qubit basis state $|01\rangle = |0\rangle \otimes |1\rangle$, where:

$$|0\rangle = \begin{bmatrix} 1 \\ 0 \end{bmatrix}, \quad |1\rangle = \begin{bmatrix} 0 \\ 1 \end{bmatrix}$$

Quantum operations (or gates) are modeled by unitary matrices *U*, which preserve the norm of quantum states. Some commonly used parameterized single-qubit gates in quantum algorithms and quantum machine learning are the rotation operators:

$$\begin{aligned} R_x(\theta )&= \begin{bmatrix} \cos \!\left( \tfrac{\theta }{2}\right) & -i \sin \!\left( \tfrac{\theta }{2}\right) \\ -i \sin \!\left( \tfrac{\theta }{2}\right) & \cos \!\left( \tfrac{\theta }{2}\right) \end{bmatrix} \\ R_y(\theta )&= \begin{bmatrix} \cos \!\left( \tfrac{\theta }{2}\right) & -\sin \!\left( \tfrac{\theta }{2}\right) \\ \sin \!\left( \tfrac{\theta }{2}\right) & \cos \!\left( \tfrac{\theta }{2}\right) \end{bmatrix} \\ R_z(\theta )&= \begin{bmatrix} e^{-i\theta /2} & 0 \\ 0 & e^{i\theta /2} \end{bmatrix} \end{aligned}$$

Measurement of a quantum state is described by a Hermitian observable *M* with spectral decomposition:

$$M = \sum _{m} m P_m$$

where $P_m$ is the projector onto the eigenspace associated with eigenvalue *m*. Upon measurement of the quantum state $|\psi \rangle$, the probability of obtaining result *m* is:

$$p(m) = \langle \psi | P_m | \psi \rangle$$

and the expectation value of *M* is:

$$E(M) = \sum _{m} m \cdot p(m) = \langle \psi | M | \psi \rangle$$

For a comprehensive introduction to quantum mechanics and quantum information theory, we refer readers to the textbook by^1^.

### Related works

Quantum circuit synthesis and optimization are long-standing challenges in quantum computing, where the goal is to generate efficient, hardware-compatible circuits that realize a given target unitary or state. Several research directions have been explored over the past two decades, spanning rule-based simplification, machine learning^16,17^ and evolutionary approaches^18,19^.

Evolutionary algorithms (EAs) have emerged as a promising family of stochastic optimizers for circuit synthesis, offering flexibility and robustness against non-convexity. Early works such as^20^ applied genetic programming to evolve simple quantum algorithms. Later studies extended this idea toward more structured representations, using gate sequences as chromosomes^21^.

To address the inherent trade-offs between conflicting design objectives-including fidelity, circuit depth, two-qubit gate count, and noise robustness–researchers have increasingly turned to multi-objective evolutionary algorithms, with NSGA-II^22^ emerging as the dominant framework. Table 1 provides a detailed comparison of key multi-objective approaches in this domain.^23^ demonstrated that a multi-objective GA can jointly optimize average fidelity, maximum fidelity, and structural cost, enabling the automatic discovery of canonical circuits such as QFT and Grover.^10^ introduced QGo, a hierarchical evolutionary compiler that partitions circuits into small blocks and reconstructs them adaptively, achieving up to 50% CNOT reduction. In another direction,^24^ integrated realistic IBM noise models into NSGA-II, highlighting the trade-off between theoretical fidelity and hardware robustness.^25^ combined reinforcement learning with NSGA-II using a UMAD mutation operator to optimize parametrized quantum circuits in noisy settings. More recently,^13^ proposed a hybrid NSGA-II framework enriched with fuzzy logic and local Broyden–Fletcher–Goldfarb–Shanno (BFGS) refinement, enabling fine-grained control of circuit depth, error, and two-qubit gate count.

Alongside evolutionary approaches, compiler-based methods form a complementary research stream. Heuristic qubit mappers such as SABRE^26^ iteratively route circuits onto hardware coupling graphs by minimizing SWAP gate overhead, achieving practical scalability on industrial-scale circuits. More recently, machine-learning-based compilers have emerged: reinforcement learning approaches such as those by Huang et al.^27^ and Pascoal et al.^28^ learn routing policies directly from hardware feedback, reducing additional gate counts by up to 12% compared to heuristic baselines. Approximation-based compilers such as QUEST^29^ further exploit circuit partitioning to produce low-CNOT approximations with bounded process distance. A recent cross-architectural survey by Zhu et al.^14^ systematically categorizes these approaches across superconducting, trapped-ion, and neutral atom platforms, highlighting that fidelity, gate count, circuit depth, and compilation time remain the four central optimization targets across all platforms. Crucially, none of these compiler-level methods incorporate multi-objective fidelity-aware optimization within an evolutionary framework, which is precisely the gap our work addresses.

Despite their effectiveness, these approaches share a critical limitation: they rely–either directly or indirectly–on full statevector or unitary matrix computation for fidelity evaluation. This reliance creates a fundamental scalability bottleneck, as memory requirements scale as $O(2^{n})$ for statevectors and $O(2^{2n})$ for unitary matrices, effectively restricting fidelity-aware optimization to circuits with fewer than 12–14 qubits.

Furthermore, existing works rarely incorporate structural decomposition strategies or matrix-level approximations to alleviate this issue, leaving a significant gap in scalable fidelity-aware quantum circuit optimization. Beyond evolutionary approaches, a complementary line of work focuses on rule-based circuit simplification at the compiler level. Nam et al.^30^ developed a set of heuristics for optimizing quantum circuits that, while effective, require at least quadratic time in the number of gates, limiting scalability to large circuits. Hietala et al.^31^ presented VOQC, a formally verified optimizer implementing these passes. More recent search-based methods such as Quartz^32^ and Queso^33^ automatically synthesize exhaustive rewrite rules but incur exponential search costs and provide no quality guarantees for large circuits. A notable recent approach by Arora et al.^15^ introduces a notion of *local optimality* for quantum circuits, achieved via a cut-and-meld algorithm (OAC). Rather than targeting global optimality–which is QMA-hard–their method guarantees that every contiguous segment of size $\Omega$ is optimal with respect to a chosen oracle optimizer. By hierarchically cutting the circuit into subcircuits, optimizing them independently, and melding the results across cuts, OAC achieves more than an order-of-magnitude speedup over VOQC while maintaining or improving optimization quality. Importantly, their efficiency analysis shows that the number of oracle calls scales *linearly* with circuit size, a property that contrasts with the exponential complexity of search-based methods and motivates segment-based decomposition strategies such as those we adopt here.

Our work directly addresses this scalability gap. Unlike compiler-based methods that target a single cost metric and assume a fixed hardware mapping^14,26^, and unlike existing multi-objective evolutionary approaches that rely on full unitary evaluation^13,25,30^, our framework combines:

- Direct matrix-based fidelity surrogates that eliminate full statevector simulation while preserving evolutionary selection pressure;
- A hierarchical block decomposition strategy inspired by graph partitioning and tensor network methods, reducing complexity from $\mathcal {O}(2^{2n})$ to $\mathcal {O}(2^{2k})$ where $k \ll n$;
- An integrated NSGA-II framework that simultaneously optimizes fidelity approximation, circuit depth, and hardware-aware gate cost across the Pareto frontier.

This formulation is orthogonal to hardware-level compilers: our method produces optimized logical circuits that can subsequently be mapped to physical hardware using tools such as SABRE^26^ or platform-specific compilers reviewed in^14^.

**Table 1:** Comparison of multi-objective approaches for quantum circuit synthesis. For reference, we also include SABRE^26^, a representative single-objective heuristic compiler, to highlight that existing hardware-level mappers do not address multi-objective fidelity-aware synthesis.

References	Optimized Objectives	Fitness function/distance	Chromosome representation	Method/specific features	Technical specificities	Innovation/key contribution
^23^	$E_{\text {avg}}, E_{\text {max}},$ gates, oracle, types	Average and maximum fidelity; structural cost	Variable-length gate sequence	Multi-objective GA	Discrete and continuous mutations; automatic gate fusion	Automatic discovery of canonical circuits (QFT, Grover) from I/O
^10^	Number of CNOTs, fidelity, noise	CNOT–fidelity correlation; noisy simulation	Set of subcircuits (blocks of 3–4 qubits)	Hierarchical compilation (QGo)	Circuit partitioning into blocks + recomposition	Scalable evolutionary synthesis with up to $50\%$ CNOT reduction
^24^	Fidelity, weighted cost ($N_1 + 10 N_2$)	Fidelity under realistic IBM FakeVigo noise	Sequential list of gates with types and angles	NSGA-II	Noisy evaluation + Pareto front	Trade-off between theoretical fidelity and hardware robustness
^25^	$-R(C)$, $\Delta (C)$, *N*(*C*)	RL reward, noise, model size	Block chain $x_i$ (rotation, entanglement, encoding, measurement)	NSGA-II + UMAD	RL in standard OpenAI environments; gradient descent	Multitask PQC architecture robust to noise
^13^	Error *E*, depth *D*, number of CNOTs $G_2$	Hilbert–Schmidt distance	Parallel layers with parameters $\theta$ and mapped qubits	NSGA-II + fuzzy logic	Adaptive mutation; gate fusion; local BFGS	Dynamic control of mutations; modular NISQ-compatible structure
^26^	Gate count, depth	SWAP count heuristic	Hardware coupling graph	Heuristic mapper (SABRE)	Look-ahead SWAP insertion; reverse traversal for initial mapping	Single-objective; no fidelity term; hardware mapping only, not synthesis

## Proposed method

This section presents our scalable fidelity-aware optimization framework. Rather than introducing a novel evolutionary algorithm, we adapt the well-established NSGA-II methodology to quantum circuit optimization by integrating two novel fidelity evaluation strategies that circumvent the exponential complexity of exact unitary computation. The key innovation lies in how fidelity is approximated and integrated into the multi-objective selection process, not in the evolutionary mechanism itself.

Designing quantum circuits for NISQ devices involves balancing conflicting objectives such as fidelity, gate cost, and depth, while operating under hardware limitations like limited qubit connectivity and noise. Traditional optimization methods struggle to scale due to the exponential cost of evaluating circuit fidelity, especially beyond 12 qubits (see Table 1).

It is worth noting that our framework targets the *logical optimization* stage of quantum compilation^14^, and is intended to operate prior to hardware-specific qubit mapping and routing. The circuits produced by our method can therefore serve as optimized inputs to existing hardware compilers, making the two approaches complementary rather than competing. Our focus on scalable fidelity evaluation is motivated by the observation that current multi-objective evolutionary methods are restricted to fewer than 12–14 qubits due to the exponential cost of unitary simulation, a bottleneck that persists regardless of the downstream compilation strategy.

Our framework consists of two tightly integrated components: (1) the core NSGA-II evolutionary loop (Section 3.1), and (2) scalable fidelity evaluation strategies designed for circuits exceeding 12 qubits (Section 3.2). Table 2 provides a systematic technical comparison of our fidelity strategies against prior work.

**Table 2:** Technical comparison of fidelity evaluation strategies.

Method	Complexity time	Complexity memory	Izable parallel-	Type circuit	Limit qubit
^23^	$O(2^{2n})$	$O(2^{2n})$	No	General	$\sim$10
^10^	$O(k \cdot 2^{2b})$	$O(2^{2b})$	Yes	Modular	$\sim$14
^13^	$O(2^{2n})$	$O(2^{2n})$	No	Ansatz	$\sim$12
Ours Method 1	$O(k \cdot 2^{2b})$	$O(2^{2b})$	Yes	Sparse	>16
Ours Method 2	$O(m \cdot 2^{2k})$	$O(2^{2k})$	Yes	Dense	>16

*Key Differences:*

- ^10^ uses fixed 3–4 qubit blocks; our Method 1 uses adaptive graph-based partitioning (Louvain + METIS/KL);
- Our methods enable GPU/multi-core parallelization.

### NSGA-II optimization framework

Our framework builds upon the classical NSGA-II methodology^22^, augmented with quantum circuit-specific refinements including parametric gate optimization, structural simplification rules, and adaptive diversity control. The following steps describe the complete optimization loop.

In the context of quantum circuit optimization, each individual represents a candidate circuit whose structure and parameters evolve according to multi-objective selection pressures. The goal is to simultaneously optimize fidelity, depth, and hardware-aware gate cost while preserving population diversity and avoiding premature convergence. The framework proceeds through the following stages:

1. *Initialization:* Each individual in the population represents a candidate quantum circuit encoded as a sequence of genes. Each gene corresponds to a quantum gate and includes its type, target qubit(s), and rotation angle if applicable:
  1 $$\begin{aligned} \text {gene} = [q_t,\;\text {gate},\;q_c,\;\theta ] \end{aligned}$$
  where $q_t$ is the target qubit, $\text {gate} \in \mathcal {G} = \{H, X, Y, Z, R_x, R_y, R_z, CX, CZ\}$, $q_c$ is the optional control qubit, and $\theta$ is a rotation angle for parametric gates. This compact gate-level encoding enables efficient manipulation via crossover and mutation. The initial population of *N* individuals is generated randomly using syntactically valid gene sequences.
2. *Evaluation:* For each circuit, compute three objectives:
  Fidelity error $$\varepsilon = 1 - \mathcal {F}$$;
  Circuit depth *D* (number of sequential gate layers);
  Gate cost $C_{\text {gate}}$, using weights such as 1 (1-qubit), 5 (CNOT), etc.
  The fidelity $\mathcal {F}$ is computed using the trace overlap between the target unitary $U_{\text {target}}$ and the synthesized unitary $U_C$:
  2 $$\begin{aligned} \mathcal {F}(C) = \frac{1}{2^n} \left| \textrm{Tr}\!\left( U_{\text {target}}^\dagger \cdot U_C \right) \right| \end{aligned}$$
  This fidelity metric is phase-invariant and commonly used to evaluate the closeness between two unitaries. A higher value of $\mathcal {F}(C)$ indicates better circuit approximation to the target^34,35^.
3. *Non-Dominated Sorting:* Sort the population into Pareto fronts using non-dominated sorting.
4. *Crowding Distance Assignment:* Within each front, assign crowding distance values to maintain diversity along the Pareto front.
5. *Selection:* Apply binary tournament selection using rank and crowding distance.
6. *Crossover:* Use one-point crossover to combine two parents into offspring. Gate sequences are split at random points and exchanged.
7. *Mutation:* Apply asymmetric mutation strategies:
  *Substitution:* Replace a gate with another random one;
  *Insertion:* Add a new gate at a random position;
  *Deletion:* Remove an existing gate.
  Mutation probabilities are tuned as $p_1> p_2 > p_3$ to control exploration vs simplification.
8. *Local Parameter Optimization:* For parametric gates ($R_X$, $R_Y$, $R_Z$), we refine angle $\theta$ using a local numerical gradient:
  3 $$\begin{aligned} \theta \leftarrow \theta + \eta \cdot \frac{\mathcal {F}(\theta +\delta ) - \mathcal {F}(\theta -\delta )}{2\delta } \end{aligned}$$
9. *Structural Refinement:* Apply gate simplification rules:
  Cancel $$X \cdot X$$, $$CX \cdot CX$$;
  Merge $$RZ(\theta _1)$$ and $$RZ(\theta _2)$$;
  Eliminate identity gates.
10. *Diversity Control:* If stagnation is detected:
  Increase mutation rate;
  Accept worse individuals with simulated annealing;
  Replace worst $\alpha \%$ with random individuals.
11. *Elitist Replacement:* Merge parent and child populations, retain the top *N* individuals by rank and diversity.
12. *Termination:* Stop when a fixed number of generations is reached or convergence is observed.

### Scalable fidelity evaluation

We propose two complementary fidelity approximation strategies, each targeting different circuit topologies. Both avoid the $O(2^{2n})$ complexity of full unitary computation while preserving sufficient ranking accuracy for evolutionary selection:

1. *Independent Block Fidelity Evaluation:* Partitions the circuit into weakly interacting sub-blocks ($k \le 6$ qubits) using graph-based community detection (Louvain method) followed by recursive bisection (METIS/KL). Each block is independently optimized and then reassembled, with highly interactive qubits strategically duplicated across blocks. Best suited for modular circuits with sparse inter-block connectivity (e.g., QAOA on low-degree graphs).
2. *Overlapping Sequential Block Evaluation (Sliding Windows):* Decomposes the circuit into overlapping windows of *k* qubits with overlap *o* (typically $k = 3$–6, $o = 1$–2). Local fidelities are computed independently for each window and aggregated via geometric product $F_{\text {global}} = \prod _{i=1}^{m} F_i$, which heavily penalizes localized mismatches. This method requires no explicit partitioning and is well-suited for densely entangled circuits where modular structure is absent.

*Critical note on fidelity interpretation:* Throughout the remainder of this section, fidelity is not interpreted as an exact physical metric but as a surrogate fitness signal guiding evolutionary selection. These surrogates are not intended to numerically match the true global trace fidelity, but to preserve relative ordering between candidate circuits in a scalable and structure-aware manner.

#### Method 1: independent block-based fidelity evaluation

**Fig. 1:**
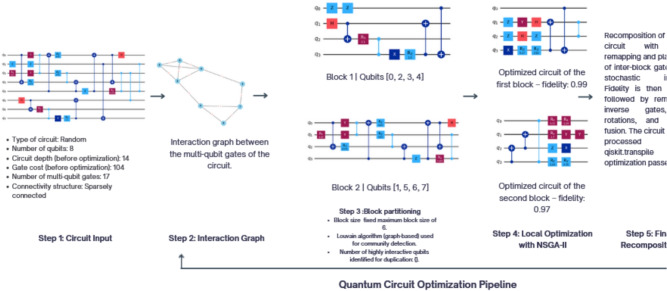
Quantum circuit optimization pipeline using block-wise fidelity evaluation.

This method avoids the $O(2^{2n})$ memory and computational cost of full unitary evaluation by exploiting circuit modularity. It partitions the circuit into weakly coupled sub-blocks based on qubit interaction patterns, optimizes each block independently via NSGA-II, and then reassembles the optimized blocks into a global circuit. Figure 1 illustrates the complete pipeline from interaction graph construction to final circuit recomposition.

The pipeline consists of seven sequential steps.

*Step 1: Quantum Interaction Graph*

A quantum circuit *C* is modeled as a weighted undirected graph $G = (V, E)$, called the *interaction graph*, where:

- each vertex $v \in V$ corresponds to a qubit,
- an edge $(i,j) \in E$ exists if a two-qubit gate (e.g., CX, CZ, RZZ) is applied between qubits $q_i$ and $q_j$,
- the weight $w_{ij}$ is the total number of such interactions.

The graph is formally defined by the adjacency matrix:

$$A_{ij} = {\left\{ \begin{array}{ll} w_{ij}, & \text {if an interaction exists between } q_i \text { and } q_j, \\ 0, & \text {otherwise}. \end{array}\right. }$$

*Step 2: Block Partitioning*

Partitioning proceeds in two phases: (a) community detection via the Louvain method^36^ to identify natural clusters of strongly interacting qubits, followed by (b) recursive bisection using hybrid METIS/KL for oversized blocks. This two-stage approach balances topological coherence with the constraint $k_{\max }$ (typically 5–6 qubits).

1. *Initial Detection Using the Louvain Method* In realistic quantum circuits, qubits do not interact uniformly; some subsets interact much more strongly. To exploit this structure, we use the Louvain community detection method^36^ on the interaction graph. The algorithm groups strongly interacting qubits into the same block, revealing hierarchical, modular structures. It follows two main phases:
  *Local Phase:* each qubit is evaluated with respect to its immediate neighbors and may be reassigned to the community that maximizes its “interaction benefit”. This encourages concentrating two-qubit gates inside the same block.
  *Aggregation Phase:* stabilized communities are merged into “super-qubits”, producing a reduced graph that is recursively reprocessed to form a multi-level hierarchy.
2. *Refinement by Recursive Bisection (Hybrid METIS/KL)* Some blocks obtained through Louvain may still exceed the allowed maximum size. To address this, we apply a recursive bipartitioning scheme: overly large blocks are subdivided using METIS, and refined when necessary using KL (Kernighan–Lin), until each block satisfies size constraints. The complete partitioning procedure is formalized in Algorithms 2 and 3. Algorithm 2 implements the recursive bisection strategy, repeatedly subdividing oversized blocks until all satisfy $|Q'| \le k_{\max }$. Algorithm 3 provides the hybrid METIS/KL refinement.

**Algorithm 1:**
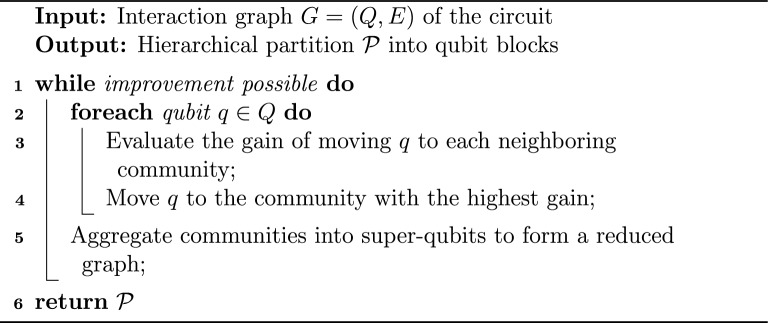
Community Detection (Louvain method adapted to qubits)

**Algorithm 2:**
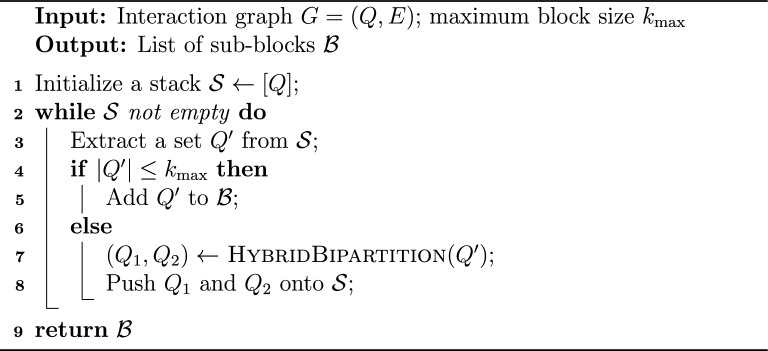
Hybrid METIS/KL Recursive Partitioning

**Algorithm 3:**
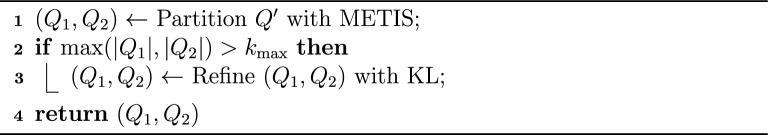
Hybrid Bipartition $$(Q')$$

*Step 3: Duplication of Highly Interactive Qubits*

Duplicated qubits correspond to symbolic placeholders used during local evaluation only. During recomposition, all placeholders are merged back into a single logical qubit, and no additional physical qubits, ancillae, or SWAP operations are introduced.

Algorithm 4 formalizes the duplication strategy. Before local block-level optimization, we identify qubits involved in many inter-block gates. These qubits may be duplicated across blocks to maintain their influence while avoiding excessive inter-block injection.

Let $q_i$ be a qubit in block $B_k$. We define:

- $g_{\text {tot}}(q_i)$: total number of multi-qubit gates acting on $q_i$,
- $g_{\text {inter}}(q_i,B_j)$: number of gates between $q_i$ and block $B_j \ne B_k$,
- $\rho (q_i,B_j) = \dfrac{g_{\text {inter}}(q_i,B_j)}{g_{\text {tot}} (q_i)}$: *inter-block interaction ratio*.

A qubit is considered highly interactive with block $B_j$ if:

$$\rho (q_i,B_j) \ge \tau \quad (e.g.,\ \tau = 0.6).$$

In this case, $q_i$ may be duplicated into $B_j$ (if size limits permit).

**Algorithm 4:**
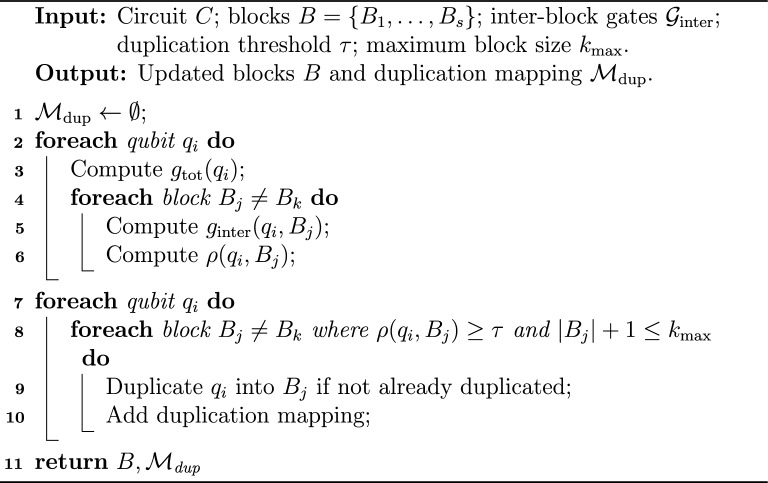
Duplicate High Interaction Qubits $(C, B, \mathcal {G}_{\text {inter}}, \tau , k_{\max })$

*Step 4: Local Multi-Objective Optimization via NSGA-II* Each extracted block is independently optimized using the NSGA-II procedure described in Section 3.1, according to the three objectives: local fidelity, depth, and gate cost. This reduces global complexity while respecting each subcircuit’s structural constraints.

*Step 5: Global Recomposition* All optimized subcircuits are then assembled into a single global circuit using recompose_from_blocks(), which preserves the gate order of the original circuit.

*Step 6: Controlled Reinsertion of Inter-Block Gates*

After recomposition, the removed inter-block gates are reintroduced in a controlled manner.

- If interacting qubits were duplicated, the duplicated version is used and the gate becomes intra-block.
- Otherwise, the gate is injected between source and target blocks, ensuring connectivity and fidelity constraints are respected.

The injection follows a fidelity-guided stochastic strategy:

$$\text {gain} = \mathcal {F}_{\text {after}} - \mathcal {F}_{\text {before}} > 0.$$

Each inter-block gate is therefore validated according to its actual contribution to global fidelity.

*Step 7: Final Compression* A final structural refinement phase is applied to the recomposed circuit, using the methods presented in (*Circuit Structural Refinement*).

#### Method 2: fidelity evaluation using overlapping sequential blocks

**Fig. 2:**
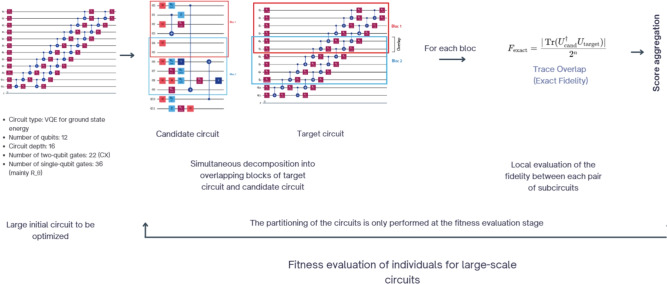
Quantum circuit optimization pipeline using overlapping block-based fidelity evaluation.

This method addresses two limitations of Method 1: (a) it does not require explicit circuit partitioning (which may be suboptimal for densely entangled circuits), and (b) it naturally handles short-range entanglement through overlapping windows, avoiding boundary artifacts. Unlike Method 1, which optimizes blocks independently, this approach uses overlapping windows solely for fitness evaluation within a global NSGA-II loop. Figure 2 illustrates the integration of sliding-window fidelity into the NSGA-II pipeline.

*Step 1: Sequential Decomposition via Sliding Windows*

The candidate circuit and the target circuit are decomposed into a series of local subcircuits, called *windows*. Each window contains a contiguous set of *k* qubits (typically $k = 3$ to 6), and consecutive windows overlap by *o* qubits (typically $o = 1$ or 2).

Thus, a circuit of *n* qubits is covered by approximately:

$$\frac{n}{k - o} \quad \text {windows.}$$

This sliding-window scheme maintains continuity of entanglement between adjacent blocks while keeping each local unitary of manageable dimension.

*Step 2: Local Fidelity Evaluation*

For each window, we extract the corresponding candidate subcircuit $U_i$ and target subcircuit $V_i$. Local fidelity is computed using the trace-overlap metric (Eq. 2), here adapted to a window of size *k*:

$$F_i = \frac{\left| \textrm{Tr}\big (U_i V_i^\dagger \big ) \right| }{2^k}.$$

The set of local fidelities $\{F_i\}$ represents the circuit quality measured at different overlapping regions.

*Step 3: Aggregation into a Global Fidelity*

Local fidelities are combined via geometric product:

4 $$\begin{aligned} F_{\text {global}} = \prod _{i=1}^{m} F_i. \end{aligned}$$

We emphasize that this aggregated quantity (Eq. 4) is not intended to approximate the numerical value of the global trace fidelity. Instead, it serves as a relative fitness signal within the NSGA-II selection process. Its multiplicative form intentionally penalizes localized mismatches–a single low-fidelity window drastically reduces $F_{\text {global}}$–and prevents poorly aligned regions from being masked by high-fidelity subcircuits elsewhere.

This aggregation has two advantages:

1. It strongly penalizes windows with low fidelity, preventing a poorly matched region from being overshadowed by well-aligned ones.
2. It remains stable even when multiple windows share qubits and introduce inter-window dependencies.

*Step 4: Global Multi-Objective Estimation*

Unlike Method 1, no independent optimization is applied within each window. Instead, NSGA-II optimizes the entire circuit globally using the sliding-window fidelity as its primary fitness signal. Each candidate circuit is evaluated using the multi-objective vector:

$$\text {Fitness}(C) = \left[ F_{\text {global}}(C),\; -d(C),\; -Cost(C) \right] ,$$

where *d*(*C*) is the circuit depth and *Cost*(*C*) is the hardware-aware weighted gate cost. This evaluation guides the NSGA-II evolutionary process across fidelity, depth, and structural efficiency.

*Step 5: Validation and Correction*

When computationally feasible (circuits $\le 14$ qubits), approximate fidelity is compared with exact fidelity. If error exceeds threshold $\epsilon$, targeted corrections (angle refinements, redundant gate removal) are applied to the most problematic windows. The entire approach is summarized in Algorithm 5.

**Algorithm 5:**
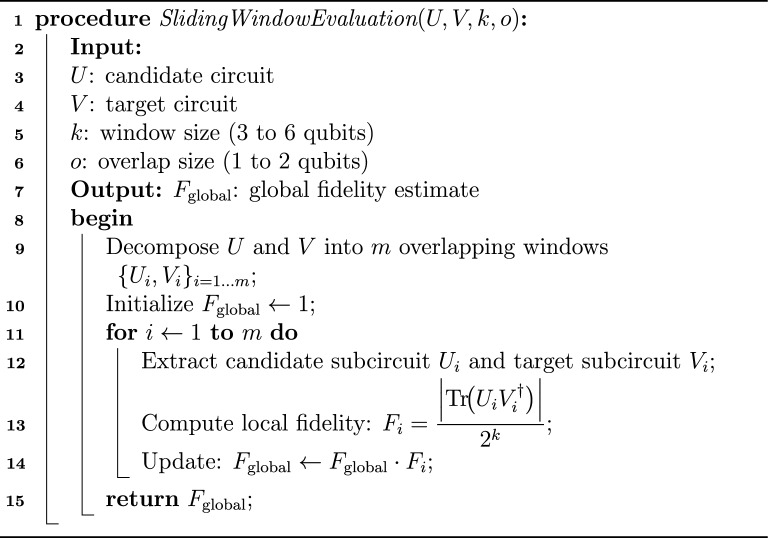
Sliding-Window Fidelity Evaluation

Both methods reduce the intractable complexity of fidelity computation from $O(2^{2n})$ to $O(2^{2k})$ (or $O(2^{2b})$), where $k, b \ll n$. Their blockwise nature enables parallel execution across CPU cores or GPU threads. This significantly extends fidelity-aware optimization beyond the limits of full-unitary simulation and enables the treatment of circuits with several tens of qubits under bounded block sizes and parallel evaluation assumptions.

## Results and discussion

All code, data, test circuits, and experimental configurations are publicly available at https://github.com/Roumy126/code_PFE, allowing full reproducibility of the results.

We conducted a comprehensive set of experiments using reference quantum circuits of various complexity levels. These circuits range in size from small (3–4 qubit) to large (15+ qubit) architectures. Our test suite includes both randomly generated circuits and manually constructed circuits representing common quantum algorithms such as QAOA, VQE, and QFT. Additionally, we incorporated well-known quantum circuits from the MQT Bench benchmark collection (https://www.cda.cit.tum.de/mqtbench/), which provides a wide variety of realistic quantum circuit instances designed to evaluate compilation and optimization techniques. These circuits were chosen to assess the performance of our method across diverse topologies and entanglement patterns. Furthermore, we designed custom test cases with high inter-qubit connectivity to stress-test the partitioning and recombination mechanisms of the algorithm. This methodology offers valuable insights into the algorithm’s scalability and adaptability when applied to complex quantum architectures.

### Algorithm configurations

All experiments use the following NSGA-II configuration, calibrated through preliminary sensitivity analysis:

- *Population size:* 200–300 individuals, scaled based on circuit depth and qubit count;
- *Number of generations:* 500–1000, with early stopping if Pareto front convergence is detected (no improvement over 50 generations);
- *Crossover probability:* Set to 0.9, using a one-point crossover mechanism;
- *Mutation rate:* Fixed at 0.1;
- *Selection strategy:* Binary tournament selection with crowding distance to ensure population diversity.
- *Statistical methodology:* All reported results are averaged over **10 independent runs** with different random seeds. For each configuration, we report the mean fidelity, depth, gate count, and cost across these runs. The variability across runs was consistently low (standard deviation below 0.02 in fidelity for all reported instances), confirming the robustness of the optimization process.

The MQT Bench circuits used in our evaluation represent a standardized and widely-adopted benchmark suite for assessing quantum compilation and optimization techniques^37^. This choice ensures reproducibility and comparability with related work, addressing the lack of cross-platform standardized benchmarks recently identified as an open challenge in the community^14^.

### Experimental results

Through the effective configuration of our scalable multi-objective algorithm, we achieved promising results in the automated synthesis of optimized quantum circuits. Specifically, for small-scale circuits (up to 8 qubits), the resulting fidelity consistently exceeded 0.97. For medium-scale circuits (10–16 qubits), approximate fidelity values ranged between 0.85 and 0.90 using block-based evaluation. For large-scale circuits (32 qubits), approximate fidelity reached 0.81; while below the ideal threshold, this result is obtained in a regime where exact evaluation is entirely infeasible, making it a viable operating point for NISQ-oriented design exploration. These results were accompanied by a significant reduction in both circuit depth and implementation cost.

For large-scale circuits (typically beyond 12 qubits), the overall fidelity is estimated using a weighted aggregation of local block fidelities, as defined in Equation 2. To assess the accuracy of this approximate measure, we compared its value with the exact fidelity computed via the trace formula 2, whenever feasible (i.e., for circuits ranging from 10 to 14 qubits). The results demonstrate a strong agreement between the two approaches, with negligible discrepancies. This empirical validation allows us to confidently extend the use of the aggregated fidelity to larger circuits (beyond 16 qubits), where exact fidelity becomes inapplicable in practice due to memory limitations imposed by the exponential growth of matrix size. Therefore, the aggregated fidelity serves as a reliable and scalable metric for evaluating the quality of the generated quantum circuits.

In the following sections, we present the detailed results obtained from various case studies. For each scenario, we report both the characteristics of the original quantum circuits and those of the optimized counterparts. The experiments aim to evaluate the trade-off between accuracy, structural cost, and computational efficiency.

### Fidelity evaluation using independent blocks

#### Experiments on random circuits

The first series of tests applies the independent-block evaluation method to random quantum circuits, in order to assess its robustness in non-problem-specific configurations. The key idea is to partition a complex circuit into weakly connected subsets of qubits, optimize each sub-block separately, and then reconstruct the global circuit. This approach reduces the circuit depth and number of gates while providing an approximate estimate of global fidelity.

The results summarized in Table 3 highlight that the independent-block approach achieves substantial depth and gate reductions while maintaining a fidelity approximation close to the exact reference. The method proves robust even for circuits with over ten qubits. The figure below 3 illustrates the partitioning of a complex random quantum circuit into sub-ensembles of weakly connected qubits. Each block is optimized independently to reduce depth and gate count before reconstructing the global circuit to estimate the final fidelity.

**Fig. 3:**
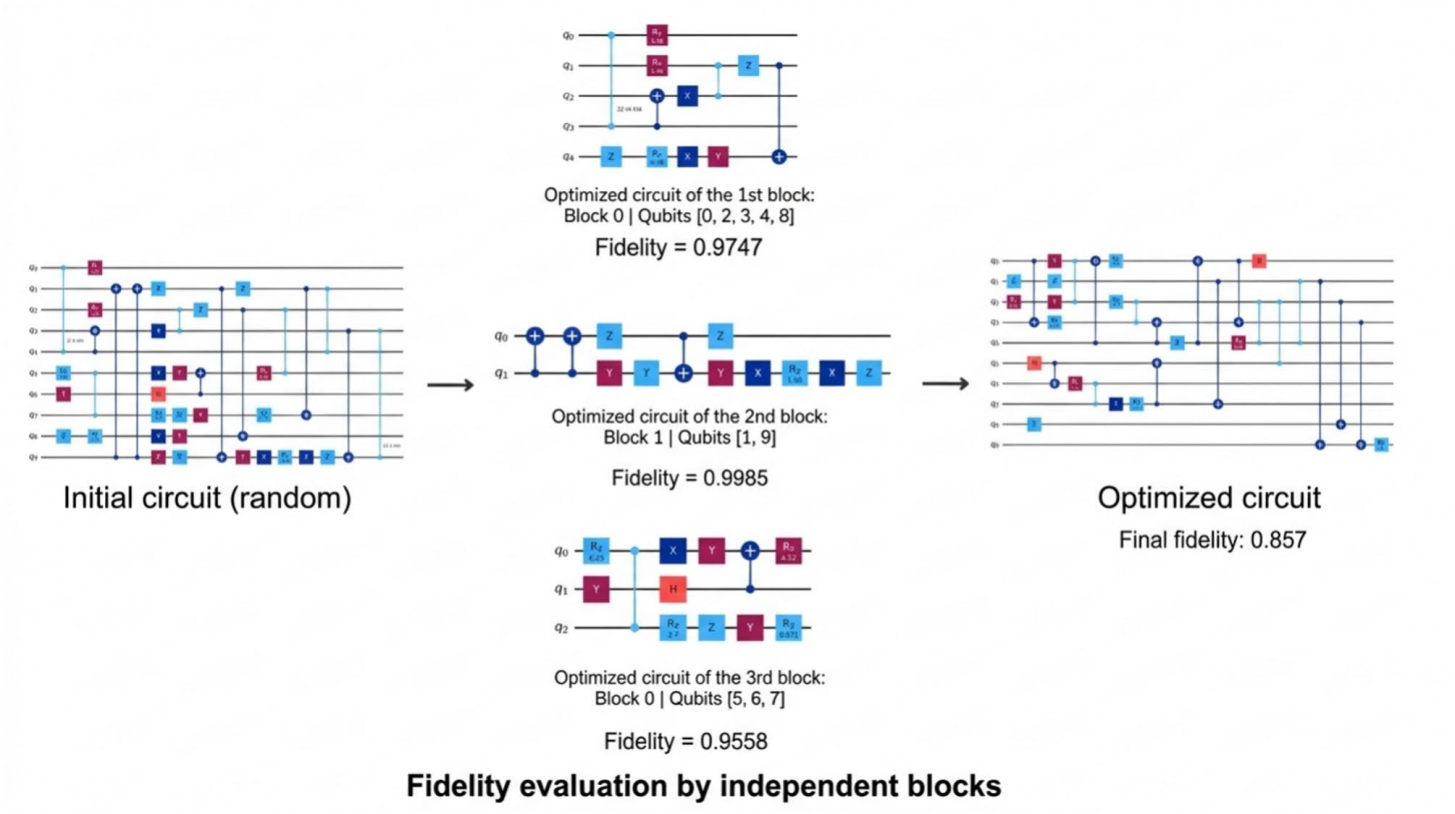
Example of circuit optimization before and after applying the fidelity evaluation by independent blocks.

**Table 3:** Experimental results on random circuits using the independent-block fidelity estimation method. All values are averages over 10 independent runs (standard deviation in fidelity $< 0.02$ for all instances).

Test	Qubits	Approx. Fid.	Exact Fid.	Blocks	Depth		Gates		Cost		Inter-Block Gates
					Before	After	Before	After	Before	After	
1	8	0.9460	0.8960	{0,2,3,4}, {1,5,6,7}	11	7	30	24	87	63	2
2	8	0.9600	0.9460	{0,2,4,5}, {1,3,6,7}	13	8	35	26	96	81	4
3	10	0.8977	0.8577	{0,2,3,4,8}, {1,9}, {5,6,7}	14	10	56	40	104	76	2
4	12	0.86	0.81	{0,2,3,4,8,9}, {1,5,6,7,10,11}	13	10	58	50	98	80	1
5	16	0.87	/	{0,3,4,5,7,9}, {1,6,10,12,14}, {2,8,11,13,15}	9	8	60	40	221	120	3
6	32	0.81	/	6 blocks	20	12	76	38	282	190	1

*Note on exact fidelity availability:* For Tests 5 and 6 (16 and 32 qubits respectively), the exact unitary fidelity is not reported (marked “/”) because constructing the full $2^n \times 2^n$ unitary matrix exceeds available memory on our experimental hardware (16 GB RAM). The approximate block-based fidelity is therefore the sole quality measure for these instances, consistent with the scalability regime targeted by our method.

To analyze the optimization behavior of the proposed method, we evaluate several multi-objective performance indicators during the evolutionary process. In particular, we monitor the Hypervolume (HV), Spacing, and Spread indicators for each circuit block independently. These indicators allow us to assess the convergence, diversity, and distribution quality of the Pareto front obtained during the optimization.

The Hypervolume (HV) measures the dominated portion of the objective space and reflects the convergence quality of the solutions. The Spacing indicator evaluates the uniformity of the distribution of solutions on the Pareto front, while the Spread indicator measures the diversity and extent of the obtained solutions.

**Fig. 4:**
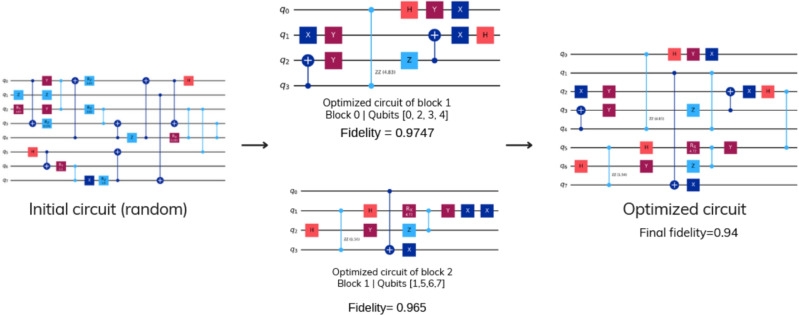
Example of circuit optimization before and after applying the fidelity evaluation by independent blocks.

**Fig. 5:**
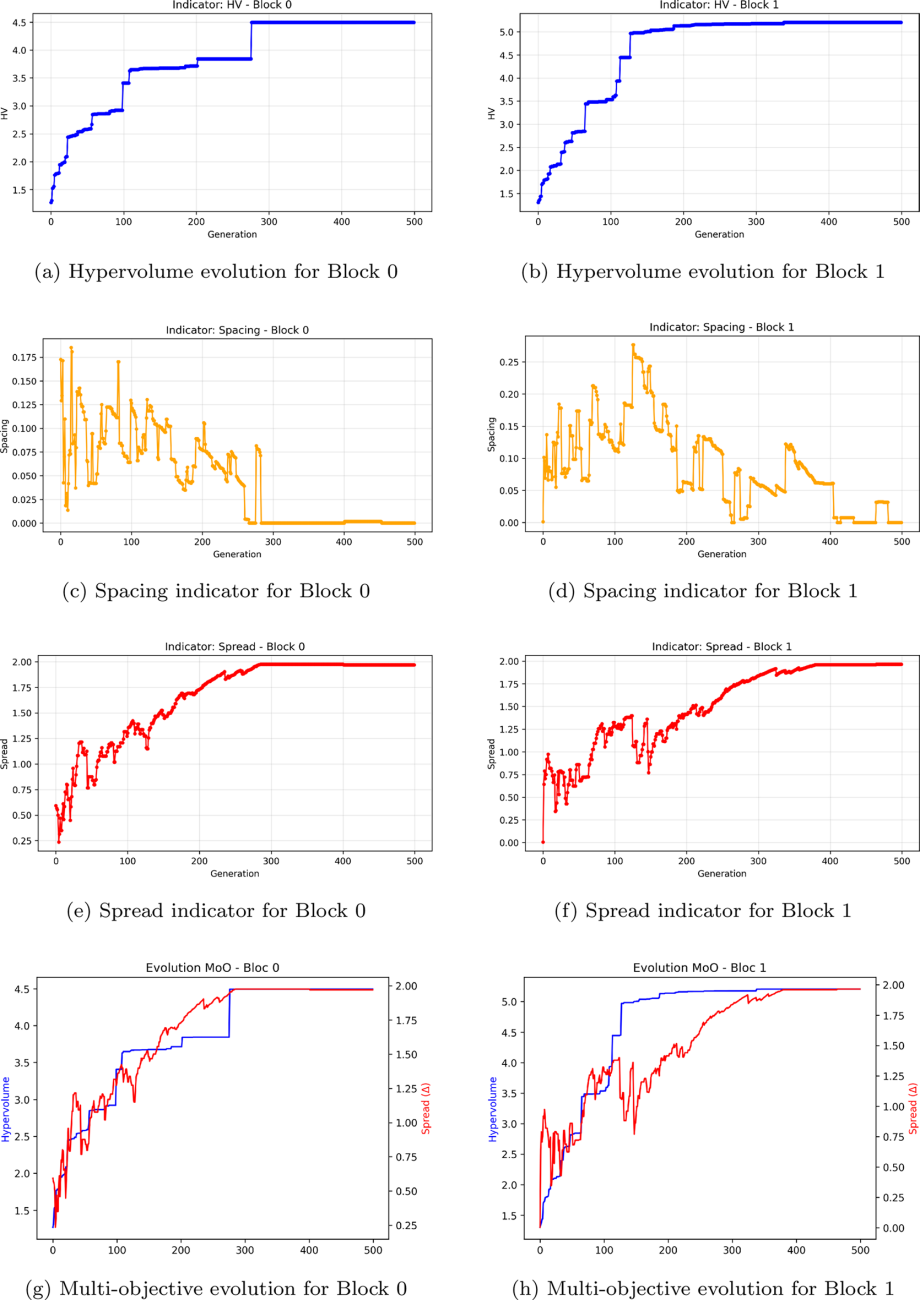
Evolution of multi-objective optimization indicators during the evolutionary process for the different circuit blocks. The Hypervolume (HV) measures convergence quality, the Spacing indicator evaluates the uniformity of solution distribution, and the Spread indicator reflects the diversity of the Pareto front. The last row shows the joint evolution of objectives during the multi-objective optimization process.

Figures  4 and 7 illustrate the circuit optimization process and the evolution of these indicators for the different blocks during the evolutionary search (Fig. 5).

#### Experiments on problem-oriented circuits

We also applied the independent-block method to problem-oriented quantum circuits, such as QAOA circuits on chain or low-degree graphs, and simplified VQE ansätze. In these cases, qubit interactions are naturally limited by the problem’s topology, making the decomposition into blocks particularly suitable.

The results in table 4 show that, for such circuits, the approach significantly reduces both circuit depth and cost while preserving a close approximation of the overall fidelity $\mathcal {F}$. Furthermore, local evaluation enables parallel computation of subcircuits, yielding substantial runtime savings.

**Table 4:** Experimental results on problem-oriented circuits using the independent-block fidelity estimation method. All values are averages over 10 independent runs (standard deviation in fidelity $< 0.02$).

Circuit	Qubits	Approx. Fid.	Exact Fid.	Blocks	Depth		Gates		Cost		Inter-Block Gates
					Before	After	Before	After	Before	After	
QAOA	8	0.9460	0.8960	{0,2,3,4}, {1,5,6,7}	11	7	130	98	87	63	2
QAOA	14	0.8955	0.8460	{0,1,3,4,5,12;13}, {2,6,7,8,9,10,11}	14	11	180	130	107	87	3

#### Qualitative comparison with exact evaluation

Compared with the exact (non-partitioned) simulation, the independent-block approach introduces a limited loss in precision (typically 5–$10\%$ in fidelity). However, it achieves significant savings in both time and memory, reducing circuit depth by up to $40\%$. The method thus enables the manipulation of circuits that would otherwise be intractable under exact simulation. This trade-off between computational cost and accuracy is particularly favorable for intermediate-size circuits and aligns well with NISQ constraints, where resources are limited. Overall, the method maintains coherent fidelity estimation beyond 10 qubits while offering superior scalability.

### Overlapping sliding-windows evaluation

#### Experiments on random circuits

The analysis of the results in Table 5, obtained from random circuits, shows that the overlapping sequential block evaluation–integrated into NSGA-II–achieves a significant reduction in resource usage (depth reduced by half, 30–40% fewer gates, and up to 50% lower cost), while maintaining high fidelity ($\ge 0.85$). The discrepancy between the approximate and exact fidelities remains below 0.05, confirming the robustness of the approach. Beyond these structural improvements, the method also provides a substantial computation-time gain, making the evaluation scalable for medium- to large-scale circuits, where exact estimation would be intractable.

**Fig. 6:**
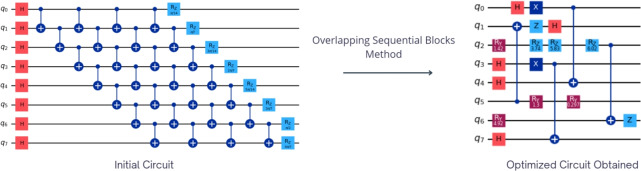
Example of circuit optimization before and after applying the overlapping sliding-window method.

To provide a concrete illustration of the structural transformations induced by the overlapping sliding-window optimization, Fig. 6 shows a representative example of a quantum circuit before and after the optimization process.

**Table 5:** Experimental results on random circuits using the overlapping-block (sliding-window) fidelity estimation method. All values are averages over 10 independent runs (standard deviation in fidelity $< 0.02$).

Instance	Qubits	Approx. Fid.	Exact Fid.	Depth		Gates		Cost	
				Before	After	Before	After	Before	After
1	8	0.9623	0.9102	16	8	38	24	93	58
2	11	0.8707	0.8298	14	9	42	31	127	87
3	10	0.9112	0.8456	21	7	52	19	218	57
4	20	0.9036	/	9	4	70	28	210	41
5	30	0.8614	/	28	14	95	47	312	143

#### Experiments on problem-oriented circuits

The analysis of Table 6 confirms that, as in the case of random circuits, the overlapping block evaluation method integrated into NSGA-II remains effective on problem-oriented circuits. In the example of the 9-qubit VQE circuit, we observe a significant reduction in depth (from 13 to 8), number of gates (from 53 to 36), and overall cost (from 107 to 69). The approximate fidelity (0.8973) remains close to the exact fidelity (0.8712), with only a small deviation, which further confirms the robustness of the approximation.

**Table 6:** Experimental results on problem-oriented circuits using the overlapping-block (sliding-window) fidelity estimation method. All values are averages over 10 independent runs (standard deviation in fidelity $< 0.02$).

Circuit	Qubits	Approx. Fid.	Exact Fid.	Depth		Gates		Cost	
				Before	After	Before	After	Before	After
VQE	9	0.8973	0.8712	13	8	53	36	107	69
QFT	10	0.8314	0.7931	15	9	75	56	162	89

**Fig. 7:**
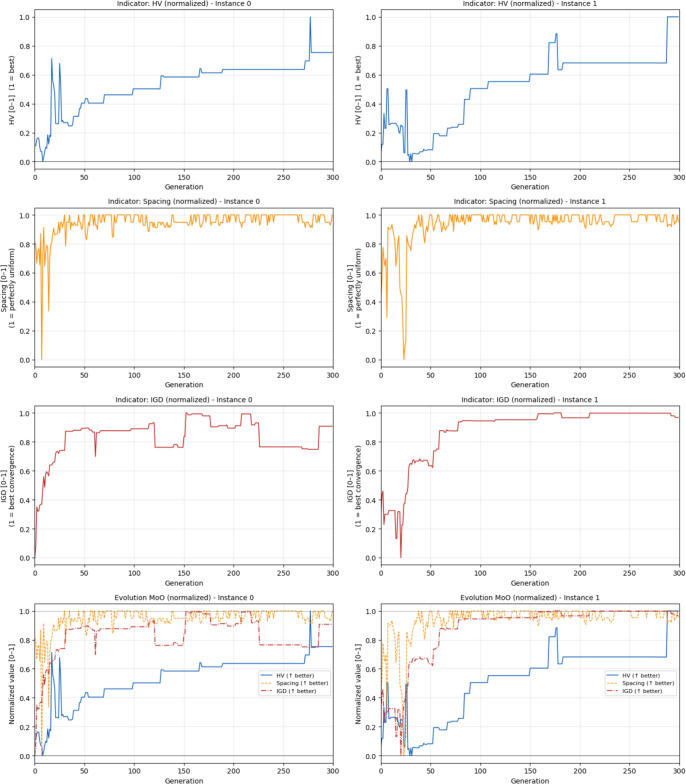
Evolution of normalized multi-objective quality indicators (Hypervolume, Spacing, and IGD) over 300 generations of the proposed NSGA-II algorithm with overlapping sliding windows evaluation, evaluated on two benchmark quantum circuit instances.

### Analysis of multi-objective indicators

Figure 7 shows that the Spacing stabilizes near 0.95–0.98 after 50 generations, confirming early diversity preservation by the method. Both HV and IGD converge monotonically toward 1.0 by generation 290, validating progressive improvement of the Pareto front quality. A transient collapse observed in Instance 1 around generations 20–40 is attributed to genetic drift in the more complex search space, followed by full recovery.

#### Qualitative comparison with exact evaluation

The classical NSGA-II approach, which relies on computing the full unitary operator, provides exact fidelity but has exponential time complexity $\mathcal {O}(2^n)$ and memory complexity $\mathcal {O}(4^n)$. This makes it intractable beyond 10–12 qubits.

In contrast, the overlapping-window approach reduces the complexity to

$$\mathcal {O}(k \times 2^b) \;\; \text {in time and} \;\; \mathcal {O}(k \times 4^b) \;\; \text {in memory},$$

where *k* is the number of windows and *b* their size. This makes the method applicable to circuits exceeding 20 qubits.

Although this approximation introduces a moderate accuracy loss (5–15%), it yields substantial depth reductions (30–40%), better scalability, and improved compatibility with NISQ constraints such as limited connectivity, noise, and short coherence times. Thus, the precision–cost trade-off becomes favorable beyond roughly 10 qubits, making the block-based approach an efficient alternative for multi-objective circuit optimization.

### Execution time comparison

An empirical comparison of execution times was conducted to quantify the computational gains achieved by the scalable fidelity estimation methods relative to the exact matrix-based evaluation.

**Fig. 8:**
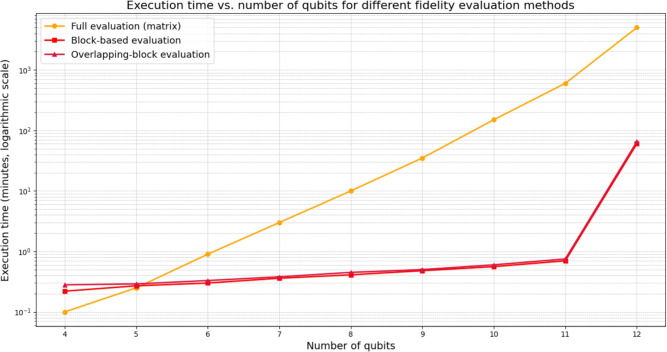
Execution time comparison between global unitary computation and scalable fidelity estimation methods (block-wise and overlapping).

As shown in Fig. 8, the classical unitary-based fidelity evaluation becomes infeasible beyond 10 qubits, exhibiting exponential growth in execution time. In contrast, the proposed block-based methods maintain near-linear scalability due to localized and parallelized evaluations.

At 14 qubits, these methods provide over a $10\times$ speed-up, enabling fidelity estimation for large circuits within reasonable computation time, even on classical hardware. These results confirm the practical advantage and scalability of the proposed approximation strategies.

Our block decomposition strategies share a conceptual motivation with the cut-and-meld algorithm (OAC) proposed by Arora et al.^15^, which hierarchically cuts a circuit into subcircuits, optimizes each segment independently via an oracle, and recomposes them while guaranteeing local optimality. Their formal analysis demonstrates that the number of oracle calls scales linearly with circuit size–a property consistent with the near-linear scalability we observe empirically with our block-based methods (Fig. 8). However, a key distinction lies in the optimization objective: OAC targets a single additive cost function (e.g., gate count) and relies on an external oracle optimizer, whereas our framework integrates fidelity estimation directly into a multi-objective NSGA-II loop, enabling simultaneous Pareto optimization of fidelity, depth, and gate cost without requiring global unitary computation. This makes our approach complementary to compiler-level methods: where OAC excels at provably optimal segment reduction, our framework addresses the scalability of fidelity-aware multi-objective synthesis beyond 12 qubits.

## Conclusion and future works

This work explores a scalable, fidelity-aware evolutionary framework for quantum circuit optimization, explicitly targeting the regime where exact unitary-based evaluation becomes infeasible. By incorporating two scalable fidelity estimation strategies–block-wise evaluation based on graph partitioning and overlapping block-based decomposition–we overcame the limitations of full unitary fidelity computation. These methods significantly reduce memory and time complexity while preserving accurate fidelity assessment.

Integrated within a structured NSGA-II pipeline, our framework optimizes three key objectives simultaneously: fidelity, gate cost, and circuit depth. Experiments on benchmark circuits from 5 to 32 qubits demonstrated that our approach consistently improves circuit quality, achieving fidelity above 0.94 for small circuits (up to 8 qubits), above 0.85 for medium-scale instances (10–16 qubits), and above 0.80 for large-scale circuits (32 qubits), along with up to $45\%$ depth reduction and substantial gate count savings, all without relying on full matrix computation.

As future work, we plan to extend this framework in several directions:

- Integrate hardware-aware noise models specific to superconducting, trapped-ion, and neutral atom platforms^14^, and validate the optimized circuits on real quantum hardware such as IBM Eagle or IonQ systems.
- Generalize the framework to support constrained circuit synthesis under QPU-specific connectivity graphs, incorporating hardware-level coupling constraints as additional objectives within the NSGA-II loop, complementing existing qubit mapping tools such as SABRE^26^.
- Investigate hybridization with reinforcement learning-based routing strategies^27,28^ to jointly optimize logical fidelity and physical mapping cost in a unified pipeline, moving toward an end-to-end hardware-aware circuit synthesis framework.

Our results demonstrate that evolutionary methods–when paired with scalable and structure-aware fidelity evaluation–can unlock practical and efficient circuit design for next-generation quantum applications.

## Data Availability

Publicly available in a repository: https://github.com/Roumy126/code_PFE
